# A cross-sectional, facility based study of comorbid non-communicable diseases among adults living with HIV infection in Zimbabwe

**DOI:** 10.1186/s13104-016-2187-z

**Published:** 2016-08-02

**Authors:** Itai M. Magodoro, Tonya M. Esterhuizen, Tawanda Chivese

**Affiliations:** 1 IMM, Department of Interdisciplinary Health Sciences, Faculty of Medicine and Health Sciences, Stellenbosch University, PO Box 241, Cape Town, 800 South Africa; 2 Department of Interdisciplinary Health Sciences, Faculty of Medicine and Health Sciences, TME, Centre for Evidence Based Health Care, Stellenbosch University, PO Box 241, Cape Town, 800 South Africa; 3 Department of Interdisciplinary Health Sciences, Faculty of Medicine and Health Sciences, TC, Centre for Evidence Based Health Care, Stellenbosch University, PO Box 241, Cape Town, 800 South Africa; 4 Department of Medicine, Faculty of Health Sciences, Chronic Disease Initiative for Africa, University of Cape Town, Observatory, 7925 South Africa

**Keywords:** HIV, Non-communicable diseases, Comorbidity, Multimorbidity, Sub-Saharan Africa

## Abstract

**Background:**

Increased antiretroviral therapy uptake in sub-Saharan Africa has resulted in improved survival of the infected. Opportunistic infections are declining as leading causes of morbidity and mortality. Though comprehensive data are lacking, concern has been raised about the rapid emergence of non-communicable diseases (NCDs) in the African HIV care setting. We therefore set out to characterise the NCD/HIV burden among adults living and ageing with HIV infection in Zimbabwe.

**Methods:**

We conducted a cross-sectional study among patients receiving care in a public sector facility. We reviewed patient records and determined the prevalence of comorbid and multi-morbid NCDs. Associations with patient characteristics were evaluated using univariate and multi-variate logistic regression modelling. Significance testing was done using 2-sided p values and 95 % confidence intervals calculated.

**Results:**

We recruited 1033 participants. 31 % were men. Significant gender differences included: older median age, more advanced disease at baseline, and greater use of stavudine and protease inhibitor containing regimens in men compared to women. The prevalence of comorbidity and multi-morbidity were, respectively, 15.3 % (95 % CI 13.3–17.7 %) and 4.5 % (95 % CI 3.4–6.0 %). Women had higher rates than men of both co-morbidity and multi-morbid

ity: 21.8 vs. 14.9 %; p = 0.010 and 5.3 vs. 2.9 %; p = 0.025 respectively. The commonly observed individual NCDs were hypertension [10.2 %; (95 % CI 8.4–12.2 %)], asthma [4.3 % (95 % CI 3.1–5.8 %)], type 2 diabetes mellitus [2.1 % (95 % CI 1.3–3.2 %)], cancer [1.8 % (95 % CI 1.1–2.8 %)], and congestive cardiac failure [1.5 % (95 % CI 0.9–2.5 %)]. After adjusting for confounding, only age categories 45–≤55 years (AOR 2.25; 95 % CI 1.37–3.69) and >55 years (AOR 5.42; 95 % CI 3.17–9.26), and female gender (AOR 2.12; 95 % CI 1.45–3.11) remained significantly and strongly associated with comorbidity risk.

**Conclusions:**

We found a substantial burden of comorbid non-communicable diseases among HIV infected patients in a high HIV and low-income setting. Integrating non-communicable diseases care, including active screening, with HIV care is recommended.

## Background

The successful rollout of highly active antiretroviral therapy (HAART) has resulted in increased life expectancy of HIV infected people. However, new clinical and public health problems are emerging. Opportunistic infections and AIDS-defining cancers (ADC) which characterised the pre-HAART era have given way to the new challenges of comorbid non-communicable diseases (NCD) in those living and aging with HIV [1]. The health and life quality gains made against HIV/AIDS are now under threat from heart disease, diabetes, stroke, kidney and liver disease, (non-AIDS defining) cancers, and mental health problems which are increasingly prevalent and documented among the HIV positive [2]. For example, cardiovascular disease has emerged as the leading cause of morbidity and mortality in HIV infected persons in developed country settings [3].

Inevitable age-related degenerative changes (possibly accelerated in HIV infection), cumulative exposure to antiretroviral drug toxicities, effects of ongoing inflammation, progressive immune dysfunction, and long-term infection by the virus itself are postulated to drive these NCDs in HIV [4–6]. Much of what is known about HIV-NCD comorbidities has emerged from studies in high income countries (HICs) [7–11]. However, the available sparse evidence suggests similar trends of increasingly prevalent NCDs among HIV infected populations in low- and middle-income countries (LMICs), including sub-Saharan Africa [12, 13].

Zimbabwe's disease terrain, similar to most SSA countries, was until recently dominated by HIV/AIDS. There is growing recognition, however, of the contribution of NCDs to the morbidity and mortality burden in the general population. Available limited data indicate that hypertension accounts for at least 40 % of all hospital out-patient consultations [14], while cardiovascular diseases are the fourth leading cause of in-patient hospital mortality [15].

The only nationally representative survey [16] to date (2005), based on the WHO STEPwise Approach, reported modest prevalence of modifiable NCD risk factors. Hypertension and diabetes mellitus rates, for example, stood at 18 and 3.7 %, respectively. With regards to the HIV infected population, the burden of comorbid NCDs remains unknown. This is despite the fact that HIV prevalence currently stands at 15 % in a population of 14 million [17].

Lack of data notwithstanding, the potential impact of NCD comorbidity in HIV is likely to be profound in the LMICs, particularly sub-Saharan Africa (SSA), which is home to 80 % of the global 35 million people living and aging with HIV [18]. There remain huge knowledge gaps about the epidemiology, diagnosis thresholds and optimal clinical management strategies of comorbid NCDs in HIV infection in the LMIC context. Similarly, there is little data on models of care appropriate to this phenomenon of colliding epidemics—NCDs and infections—occurring against the backdrop of fragile health systems and severe resource constraints [19]. Consequently, the evidence base to critically inform interventions and strategies to effectively meet these new and rapidly growing challenges in SSA is thin. Understanding the NCD burden among those living and ageing with HIV is critical if the gains already made in the fight against HIV are to be maintained.

As an initial step in mapping the NCD-HIV comorbidity terrain in low income, high HIV burden settings, the study sought to describe the prevalence and patterns of non-communicable disease among adults living with HIV infection in Zimbabwe. Additionally, the study investigated potential associations between prevalence and patterns of comorbidities with patient characteristics like HIV disease severity markers and exposure to different ART drug classes.

## Methods

### Study setting

The study was conducted at the Mpilo HIV Clinic, an out-patient facility of Mpilo Central Hospital, a tertiary care and university teaching hospital in Bulawayo, southern Zimbabwe. The clinic has been offering both adult and paediatric HIV care including antiretroviral therapy (ART) since 2004. It serves as a referral facility for other primary care facilities in Bulawayo and also accepts walk-in patients. Notably, routine care in the Clinic does not include active screening for non-communicable diseases. As a public sector facility, there are no user-fees for patients. To date, Mpilo HIV Clinic has nearly 12,000 registered adult and paediatric patients with approximately 8200 being actively followed up.

Registered general nurses with additional HIV specialist training provide the bulk of ongoing clinical care. Doctors provide complementary care on consultation basis, with diagnostic support from the hospital's laboratory and radiology services. Patient information is stored in electronic and paper-based files, and is generally linkable through unique patient identifier numbers. Electronic records are based on FUCHIA software (Epicentre, Paris, France). Patient data on HIV disease stage, incident AIDS related and non-AIDS illnesses, prescribed drugs and laboratory test results are recorded at each clinic visit.

The clinic's patient population is drawn from Bulawayo, Zimbabwe's second largest city. It is an urban setting with a population of 800,000 and is predominantly Black African. Bulawayo's HIV prevalence during the period under review was 22 %, contrasted with the national rate of 15 % [17]. Zimbabwe, typical of other low-income countries undergoing transition, has an emerging epidemic of NCDs with available data suggesting high prevalence of modifiable NCD risk factors in the general population [16].

### Study design

We conducted a cross-sectional study among patients receiving routine care for HIV infection. The study was based on records review with files sampled after patients' consultations. Data on demographics, HIV disease severity markers, and antiretroviral drug history are electronically filed. Past medical history and current illnesses including communicable and non-communicable diseases are recorded in paper-based case files. Both data bases, that is, the electronic and paper-based records, are immediately updated after every clinic visit by the patient.

Using a pre-designed and pilot tested data collecting instrument, each patient's files were systematically reviewed to detect their NCD status. Detailed clinical information on how individual NCD cases were diagnosed was not available, and hence case ascertainment was not possible.

Files of all patients presenting consecutively to the study facility for clinical review and/or drug re-supply during the study period (September/October 2015) were eligible for review. Inclusion criteria were age of at least eighteen (18) completed years at time of enrollment into the study and receiving ART at Mpilo HIV Clinic. Only patients registered with Mpilo HIV Clinic were eligible for recruitment. This is because their complete medical records, both electronic and paper-based, could be easily accessed from the Clinic compared to non-registered patients. Infected pregnant and lactating women do not receive their HIV care in the clinic as they attend separate Prevention of Mother to Child Transmission of HIV (PMTCT) facilities and were thus automatically excluded from the study.

### Sample size and data analysis

Based on anecdotal evidence, we estimated that the prevalence of co-morbidity in this population was 15 %, and we stated a required precision of 4 % (width of full confidence interval). A sample size of 1, 225 was calculated to yield a 95 % confidence interval of 0.13–0.17 around a prevalence of 0.15. This was considered adequate precision for the study. Descriptive analyses using percentages, frequencies, and tabulation were undertaken. Prevalence of comorbidity and multi-morbidity were reported as proportions with 95 % confidence intervals.

Age-distribution of comorbidity/multi-morbidity were explored using the age categories 18–≤35, 36–≤45, 46–≤55, and >55 years, with stratification by sex, CD4+ cell counts (<350 and ≥350), and ART drug class (protease inhibitor (PI) and non-PI exposure) using the x^2^ test. CD4+ cell counts nearest study end date (31 October 2015) were used, while co-morbidity patterns across the individual chronic diseases were also investigated.

Univariate significance testing for factors associated with co-morbidity, was done using x^2^ tests, Wilcoxon rank sum tests and Kruskal–Wallis tests as appropriate, as well as univariate logistic regression. Two 2-sided p values and 95 % confidence intervals were calculated. The assumption of linearity in the logit model was tested for continuous predictors using lowess curves, and where the assumption was violated, continuous variables were categorized. Multivariable logistic regression analysis was used to adjust for confounding, using a backward stepwise model with likelihood ratio tests. Categorical missing data were coded as missing and included in the model to ensure a complete sample size. All data were analysed using STATA 12.0 (StataCorp, College Station, TX, USA).

### Study variables

The study's main outcome variable was the prevalence of comorbid and multi-morbid non-communicable diseases among HIV infected adults. Comorbidity was defined as the occurrence of one non-communicable disease while multi-morbidity was the occurrence of at least two non-communicable diseases. Non-communicable diseases of study interest were diabetes mellitus, hypertension, stroke, congestive cardiac failure, cancer, renal impairment, asthma and chronic obstructive pulmonary disease (COPD).

The study also aimed to describe the distribution of these comorbidities and multi-morbidities according to additional variables including age (18–≤35, 36–≤45, 46–≤55, >55 years), sex, CD4+ cell counts (≤350 and >350 cells/ml), WHO HIV disease clinical stage (I–IV), and lastly, ART drug class exposure and duration: these may have potential application in NCD comorbidity risk stratification algorithms.

## Results

### Baseline characteristics and descriptive analysis

With 1225 as the targeted sample size, a total of 1400 consecutive adult patient’s records (>18 years old) were screened for eligibility over a study period of four (4) weeks (September–October 2015). Patients meeting all inclusion criteria constituted the final study sample of 1033. Exclusions were patients not yet commenced on ART (321) and those not registered with Mpilo HIV clinic (46). As indicated in Table 1, the overall median age at study enrollment was 42 years with an interquartile range (IQR) of 36–50 years. The median ages were 46 years (IQR 39–53) for males and 41 years (IQR 35–48) for females. Study participants were predominantly female (69 %).

**Table 1:** Characteristics of study participants and population

Variable	Sample n = 1033 (100 %)	Population n = 9658 (100 %)	Male n = 316 (30.6 %)	Female n = 717 (69.4 %)	p value comparison between males and females
Sex (females), n (%)	717 (69.4 %)	6896 (71.4 %)	–	–	
Age (years), median (IQR)	42 (36–50)	40 (37–53)	46 (39–53)	41 (35–48)	<0.001
Age category (years)					
18–≤35 (n, %)	242 (23.5)	1565 (16.2)	51 (16.2)	191 (26.7)	<0.001
35–≤45	406 (39.5)	3390 (35.1)	104 (33.1)	302 (42.4)	
45–≤55	247 (24.0)	3100 (32.1)	94 (29.9)	153 (21.4)	
>55*	134 (13.0)	1603 (16.6)	65 (20.7)	69 (9.7)	
Baseline CD4 + cell count (cells/mL), median (IQR)	191 (104–310)	184 (85–279)	174 (95–269)	202 (108–312)	0.023
Baseline CD4+ cell count below 350 cells/mL number (%)	685 (84.6)	8654 (89.6)	212 (87.2)	473 (83.3)	0.153
Baseline WHO HIV clinical stage III–IV number (%)	451 (50.8)	5360 (55.5)	156 (55.5)	295 (48.8)	0.013
Duration since HIV seropositive state (years), median (IQR)	7.1 (5.4–9.2)	6.9 (5.0–9.1)	6.7 (5.1–9.3)	7.4 (5.5–9.2)	0.073
Duration of ART exposure (years), median (IQR)	5.3 (3.4–7.1)	5.3 (2.9–6.9)	5.2 (2.8–7.0)	5.4 (3.7–7.2)	0.234
Duration ART exposure (categorical) (years)					
<2, number (%):	182 (18.2)	1729 (17.9)	65 (21.1)	117 (16.8)	0.269
2–5	262 (26.1)	2501 (25.9)	77 (25.0)	185 (26.6)	
>5	559 (55.7)	5302 (54.9)	166 (53.9)	393 (56.6)	
ART regimen frequencies, (number, %)					
PI-based	48 (4.7)	425 (4.4)	17 (5.4)	31 (4.3)	0.001
D4T/3TC + EFV or NVP	624 (60.4)	5766 (59.7)	212 (67.1)	412 (57.5)	
TDF/3TC + EFV or NVP	246 (23.8)	2115 (21.9)	56 (16.1)	190 (26.5)	
AZT/3TC + EFV or NVP	115 (11.1)	1043 (10.8)	31 (9.8)	84 (11.7)	

A total of 9658 adults on ARVs were registered with Mpilo HIV clinic during the study period. Similarities in all patient demographic and clinical characteristics were observed between the study participants and the population of patients (Table 1). Notably too, the groups were similar in terms of their ART experience.

Study subjects generally presented for care and initiated ART with advanced HIV disease: 85 % of study participants had a baseline CD4+ cell count less than 350 cells/mL. More men than women had WHO stage III and IV disease (56 vs. 48 %; p = 0.013), and men had lower CD4+ cell counts (174 vs. 202, p = 0.023) at baseline. In terms of ART experience, approximately 80 % (821) of the study population had at least two (2) years of exposure and 56 % (559) had been on treatment for five (5) years or more. There were no significant gender differences with respect to ART experience (Table 1). Stavudine (D4T) containing regimens were the most commonly prescribed (624, 60.4 %) while protease inhibitor (PI)-based combinations were the least used (48, 4.7 %). Men were more likely to receive both D4T containing (67.1 vs. 57.5 %; p = 0.001) and PI-based regimens (5.4 vs. 4.3 %; p = 0.001) compared to women.

### Burden of comorbidity and associated factors

The overall prevalence rates of comorbidity and multi-morbidity (Table 2) in the study population were, respectively, 15.3 % (95 % CI 13.3–17.7 %) and 4.5 % (95 % CI 3.4–6.0 %). The commonly observed prevalent NCDs, in order of frequency, were hypertension [10.2 %; (95 % CI 8.4–12.2 %)], asthma [4.3 % (95 % CI 3.1–5.8 %)], type 2 diabetes mellitus [2.1 % (95 % CI 1.3–3.2 %)], cancer [1.8 % (95 % CI 1.1–2.8 %)], and congestive cardiac failure [1.5 % (95 % CI 0.9–2.5 %)]. Less frequently observed conditions included hepatitis B liver disease, blood dyscrasias, cardiac arrhythmias, musculoskeletal and psychiatric/mental health disorders, and these were collectively labelled as “others”.

**Table 2:** Prevalence of individual NCDs, comorbidity and multi-morbidity stratified by gender

	Overall		Males		Females		p value
	n	Prevalence (95 % CI)	n	Prevalence (95 % CI)	n	Prevalence (95 % CI)	
Comorbidity	203	19.6 (17.3–22.2)	47	14.9 (11.1–19.3)	156	21. 8 (18.8–25.0)	0.010
Multi-morbidity	47	4.6 (3.3 6.1)	9	1.8 (1.3–2.3)	38	2.7 (2.4–3.2)	0.025
Hypertension	106	10.2 (8.4–12.3)	22	7.0 (4.4–10.4)	84	11.7 (9.5–14.3)	0.020
T2DM	22	2.1 (1.3–3.2)	6	1.9 (0.7–4.1)	16	2.2 (1.2–3.6)	0.731
Asthma	45	4.3 (3.1–5.8)	7	2.2 (0.9–4.5)	38	5.3 (3.8–7.2)	0.026
Cancer	19	1.8 (1.1–2.8)	6	1.9 (0.7–4.1)	13	1.8 (1.0–3.1)	0.925
Congestive cardiac failure	16	1.5 (0.9–2.5)	3	0.9 (0.2–2.7)	13	1.8 (1.0–3.1)	0.299
Stroke	10	1.0 (0.5–1.8)	2	0.6 (0.08–2.3)	8	1.1 (0.5–2.2)	0.465
Others	39	3.8 (2.7–5.1)	9	2.8 (1.3–5.3)	30	4.2 (2.8–5.9)	0.297

The patterns and distribution of comorbidity and multi-morbidity are shown in Tables 2 and 3. Stratified by gender, women had higher rates of both comorbidity (21.8 vs. 14.9 %; p = 0.010) and multi-morbidity (5.3 vs. 2.9 %; p = 0.025) relative to the men. As shown in Table 3, there was no difference in prevalence of individual NCDs between the genders except for hypertension (11.7 vs. 7.0 %, *p* = *0.02*) and asthma (5.3 vs. 2.2, *p* = *0.*026) where these conditions were more prevalent in females than males.

**Table 3:** Prevalence of co-morbidity and multi-morbidity stratified by patient characteristics

Variable	Co-morbidity prevalence (number of events)	Multi-morbidity prevalence (number of events)
Age groups		
18–≤35 years	12.4 (30)	0.4 (1)
35–≤45 years	16.3 (66)	2.5 (10)
45–≤55 years	21.8 (54)	5.7 (14)
>55 years	38.1 (51)	16.4 (22)
p value	0.000	0.000
Gender		
Male	47 (14.9)	2.9 (9)
Female	156 (21.8)	5.4 (38)
p value	0.010	0.025
Baseline CD4+ cell counts (cells/mL)		
<350	142 (20.1)	5.0 (34)
≥350	23 (18.3)	4.0 (5)
p value	0.535	0.877
ART duration		
<2 years	20.1 (38)	4.4 (8)
2–5 years	18.3 (48)	4.2 (11)
>5 years	20.5 (115)	4.7 (26)
p value	0.730	0.88
ART regimen		
PI-based	20.4 (10)	0.0 (0)
D4T/3TC + EFV or NVP	20.3 (127)	4.8 (30)
TDF/3TC + EFV or NVP	31.7 (39)	10.7 (10)
AZT/3TC + EFV or NVP	23.5 (27)	3.5 (4)
p value	0.471	0.244

Age was another patient characteristic that demonstrated statistically significant relationship with occurrence of both comorbidity and multi-morbidity. Participants aged more than 55 years had a 38 % risk of comorbidity and a 16 % risk of multi-morbidity. These risks were three (3) and forty (40) times higher, respectively, than those in participants aged 18–≤35 years. There was no demonstrated relationship between comorbidity risk and baseline CD4+ cell counts. Similarly, neither was ART regimen nor ART duration associated with comorbidity risk (Table 4).

**Table 4:** Univariate and multivariable analyses for association between patient characteristics and comorbidity

Variable	With comorbidity (n)	Without comorbidity (n)	Univariate			Multivariable		
			OR	95 % CI	p value	AOR*	95 % CI	p value
Age (continuous) median (IQR)	46 (40–56)	41 (35–49)	1.05	1.03–1.06	<0.001			
Age category (years)								
18–≤35	30 (14.9 %)	213 (25.6 %)	1.00					
35–≤45	66 (32.8 %)	340 (41.0 %)	1.38	0.87–2.19	0.176	1.42	0.89–2.26	0.144
45–≤55	54 (26.9 %)	194 (23.4 %)	1.98	1.21–3.22	0.006	2.25	1.37–3.69	0.001
>55	51 (25.4 %)	83 (10.0 %)	4.36	2.60–7.32	<0.001	5.42	3.17–9.26	<0.001
Gender								
Male, no. (%)	47 (23.2 %)	269 (32.5 %)	1.00					
Female, no. (%)	156 (76.1 %)	560 (67.5 %)	1.59	1.12–2.28	0.010	2.12	1.45–3.11	<0.001
Baseline CD4+ cell count ≥500, no (%)	14 (6.9 %)	42 (5.05 %)	1.34	0.71–2.51	0.366			
Latest CD4+ cell count ≥500, no (%)	91 (44.8 %)	323 (38.8 %)	1.30	0.93–1.83	0.126			
Baseline WHO stage								
I	38 (21.2 %)	196 (27.4 %)	1.00					
II	49 (27.4 %)	157 (22.0 %)	1.61	1.00–2.58	0.048			
III	60 (33.5 %)	270 (37.8 %)	1.15	0.73–1.79	0.549			
IV	32 (17.9 %)	91 (12.7 %)	1.81	1.07–3.09	0.028			
Duration HIV (years) ≥7	109 (53.7 %)	354 (42.6 %)	1.59	1.14–2.22	0.006			
Duration ART (years)								
<2, no. (%)	38 (18.9 %)	145 (18.0 %)	1.00					
2–5	48 (23.9 %)	214 (26.6 %)	0.86	0.53–1.38	0.521			
>5	115 (57.2 %)	445 (55.4 %)	0.99	0.65–1.49	0.947			
ART regimen								
D4T/3TC + EFV or NVP, no. (%)	127 (62.5 %)	498 (59.9 %)	1.00					
PI-based	10 (4.9 %)	39 (4.7 %)	1.01	0.49–2.07	0.988			
TDF/3TC + EFV or NVP	39 (19.2 %)	207 (24.9 %)	0.76	0.45–1.27	0.294			
AZT/3TC + EFV or NVP	27 (13.3 %)	88 (10.6 %)	1.20	0.75–1.93	0.444			

On univariate analysis, patient characteristics demonstrating significant relationships with risk of NCD comorbidity were age (OR 1.05; 95 % CI 1.03–1.06), female gender (OR 1.59; 95 % CI 1.12–2.28), baseline WHO clinical stage IV (OR 1.81; 95 % CI 1.07–3.09) and duration of HIV infection for more than 7 years (OR 1.59; 95 % CI 1.14–2.22). However, after adjusting for confounding, only age categories 45–≤55 years (AOR 2.25; 95 % CI 1.37–3.69) and >55 years (AOR 5.42; 95 % CI 3.17–9.26), female gender (AOR 2.12; 95 % CI 1.45–3.11) remained significantly and strongly associated with comorbidity risk (Table 4).

## Discussion

The study set out to describe the prevalence of comorbidity and multi-morbidity in a population of adults living with HIV infection and receiving care at a public sector facility in Zimbabwe. This was an initial step in understanding the emergent challenges of NCD comorbidity in HIV infection. The occurrence of these comorbidities and multi-morbidities was further described according to patient demographic and clinical characteristics. It is anticipated that any observed associations with these characteristics may help in the formulation of risk stratification algorithms.

In our study, we had more female than male participants, with females presenting at a younger age and with less severe and/or less advanced HIV disease compared to their male counterparts. These findings are in keeping with sub-Saharan Africa's HIV epidemic, where transmission is largely heterosexual coupled with increased infection vulnerability of women [18], on one hand, and their better health-seeking behavior [20], on the other. We found that women were more likely to have both comorbidities and multi-morbidities, compared to men. Similarly, this pattern was observed for individual NCDs, notably more women had both hypertension and asthma, compared to men. Women also had a higher prevalence of T2DM than men though this relationship did not reach statistical significance. Though pregnant women were excluded from the study, the possible contribution of previous pregnancies to prevalent hypertension or diabetes mellitus was not investigated.

Meaningful comparisons across studies are precluded by differences in case ascertainment and the actual definitions of comorbidity. Other than findings reported from cohorts treated in high income countries [10, 11], the study by Oni et al. [21] is, to our knowledge, the only one from SSA that sought to address a similar research question as ours. In a primary care facility-based study in a peri-urban South African setting, they reported gender- and age-stratified prevalence rates of comorbidity in HIV. Amongst males, comorbidity rates were 17 % (18–34 years), 28 % (36–45 years), 34 % (46–55 years) and 20 % (>55 years). The corresponding rates among females were 28, 30, 30 and 10 %, respectively; meaning that only in the younger subjects (18–45 years) was comorbidity higher in females than males.

Within each respective gender group, our study did not further stratify comorbidity according to age groups because of very limited events. Though this limits comparison with the South African findings, our study undoubtedly suggests lower overall prevalence of HIV-related comorbidity. Noteworthy is the fact Oni et al. included only hypertension, diabetes mellitus and TB in their definition of comorbidity. They reported hypertension as the commonest comorbid condition. We had similar findings, with overall prevalence of hypertension in our study at 10.2 % (95 % CI 8.4–12.3).

To date, reported hypertension prevalence rates among HIV positive persons in SSA range from 11 % in Kenya [22] to 28 % in Uganda [23]. A recent systematic review [24] on hypertension prevalence in HIV infection and with a broader population focus reported rates ranging from 8.7 to 45.9 % in low- and middle-income countries (LMICs). Although we did not include an HIV negative comparison group in our study, our reported prevalence of 10.2 % is less than the previously reported Zimbabwean national average rate of 17.9 % in the general population [16]. Hypertension is largely asymptomatic and coupled with the clinic's lack of active screening for hypertension, it may be speculated that our reported low prevalence, relative to the general population, may represent under-diagnosis.

The literature on hypertension in HIV infected persons in SSA is sparse and methodological differences aside, findings are equivocal: some studies report higher hypertension rates in the HIV positive versus the uninfected [25] while others suggest lower prevalence or no differences [26, 27]. Evidence from a systematic review including predominantly non-African subjects concluded that the risk of hypertension among HV positive persons was higher in the ART experienced versus the ART-naïve. The review did not compare hypertension to the general population [28]. Interestingly, and in contrast to our findings, men have been reported as being disproportionately affected [21].

Our study also found type 2 diabetes mellitus (T2DM) prevalence of 2.1 % with a higher but statistically insignificant rate in females (2.2 vs. 1.9 %; p = 0.731) compared to males. We could not ascertain how the diagnosis of T2DM was made in each patient. Rates of T2DM in HIV higher than what we found have been reported elsewhere in SSA: 16 % in Kenya [29] and 23.5 % [30] in South Africa. A nationally representative survey of the general adult Zimbabwean population found T2DM prevalence of 3.7 % in 2005 [16]. This estimate (reported in 2005) is most likely outdated and unrepresentative of the current situation. Unpublished findings from an urban general population community-based survey (2012) found combined rates of self-reported prevalent T2DM and previously undiagnosed hyperglycemia (random venous blood glucose ≥11.1 mmol/l) of nearly 9 %. A more recent estimate from the WHO (2014) places T2DM (fasting glucose ≥7.0 mmol/l or on medication) prevalence at 7.8 % (95 % CI 3.0–15.9) among adults in the general population [31].

The relationship between HIV/ART and diabetes is the subject of ongoing controversy. Observations of both increased risk and no difference have been noted in high income countries [32–34]. Elevated risks are linked to, among other factors, duration of and exposure to certain ARV drugs like stavudine, efavirenz and protease inhibitors [30, 34, 35]. A systemic review (2013) of the association between HIV/ART and development of T2DM in SSA subjects implied protective effects as judged by reduced HbA1c levels [12]. Notwithstanding lack of an HIV negative comparator group within our study, and taking into consideration sample variation, our finding of a 2.1 % T2DM prevalence maybe difficult to reconcile with, firstly, the much higher background T2DM prevalence in the general population, and secondly, the high frequency of stavudine (60.4 %), efavirenz (>60 %) and protease inhibitor (4.7 %) use among study participants.

In the general population hypertension and T2DM have similar risk factors: tobacco use, unhealthy diets, physical inactivity and harmful use of alcohol. These have all been documented to be highly prevalent in some HIV positive populations [36], though they were not measured in this study. ARV drug toxicities including lipodystrophy and dyslipidemias, chronic systemic inflammation, endothelial dysfunction, coagulation disorders and the virus itself are additional risk factors peculiar to HIV disease [37].

Another finding of note was the relatively high occurrence of asthma in our study population. We report a prevalence of 4.3 % (95 % CI 3.1–5.8 %). The asthma burden in the Zimbabwean general population is estimated at 2.3 % [38]. Female study participants had higher rates than their male peers (5.3 vs. 2.2 %; p = 0.026). Though not within the scope of the study to ascertain each included diagnosis, review of some patient case notes indicated that the diagnosis of asthma in some patients was based on adult-onset wheezing, effort dyspnoea and persistent coughing. HIV trained nurses provided the bulk of on-going care including initial patient assessments. Pulmonary function testing was not available either.

Consequently, argument can be made to refer to this class of condition broadly as obstructive lung disease (OLD) [39], which includes chronic bronchitis, fixed airway obstruction, bronchial hyper-responsiveness as well as asthma. OLD has recently come to the fore as the number of persons ageing with HIV continues to grow. Known risk factors for OLD include HIV infection, pulmonary TB, tobacco smoking and indoor pollution from biomass fuel exposure [40–42]. The latter disproportionately affects women in LMICs, especially SSA. Our study only assessed history of pulmonary TB, which stood at 61 % among participants, and we did not measure the burden of the other risk factors. Asthma prevalence among HIV-infected persons is reported to be increased with possibility of a different risk factor profile from the uninfected population [43].

The study's primary outcome of HIV with any comorbid NCD has some drawbacks. From an epidemiological or health systems perspective, where the concern might be burden of care and health resources allocation, the study's end-point has currency. However, from a pathogenesis-clinical standpoint in the given contest of HIV and ART exposure, the prominent limitation is that this end-point is potentially heterogeneous. This is because whereas cardiometabolic diseases like diabetes mellitus and hypertension may have common risk factors, other NCDs like asthma arguably have a different risk factor profile, as outlined above.

Other co-variables of study interest were ART exposure, age and HIV disease severity. Overall, the study population had considerable ART experience with at least half the participants having been on treatment for at least 5 years. However, we found no significant relationships between either ARV drug class or duration of exposure with risk of comorbidity. This is in contrast with the accumulated evidence linking, for example, PIs with diabetes and cardiovascular events, or NRTIs with cardiac disease [12, 27, 28, 30]. CD4+ cell counts (baseline and latest) and baseline WHO stage had no demonstrated significant associations with comorbidity risk. Similarly, duration of (known) HIV sero-positive state was insignificant after multi-variate analysis. All these factors have been previously linked with comorbidity development [44]. Our study may have been underpowered to detect these effects because of its relatively small sample size.

Lastly, the HIV epidemic in SAA is maturing with ever growing numbers of adults ageing with HIV, notwithstanding the observation from this study of a relatively small proportion (13 %) being aged 55 years or more. In the general population, NCD risks are known to be considerably increased in this age (50+ years old) group. Indeed, our findings point to age as being an important risk factor for both comorbidity and multi-morbidity in the HIV infected population. In multi-variate analysis adjusting for gender, duration of HIV sero-positive state, ART regimens received, and baseline WHO HIV disease stage and CD4 + cell counts, each additional year of age was associated with a 6 % increased risk of comorbidity (adjusted OR 1.06; 95 % CI 1.04–1.08).

### Limitations

A number of limitations with our study warrant cautious interpretation of its findings. Firstly, as a health-facility based study, our inferences about the burden of comorbidity in the wider HIV-infected Zimbabwean adult population can only be guarded. A community-based study would have been ideal. Related to this is the second limitation: selection bias. The study site is an HIV out-patient service of a tertiary care and teaching hospital, which also runs specialist medical out-patient clinics. It is likely that HIV positive patients with more severe comorbid NCDs were preferentially reviewed in the medical clinics inadvertently reducing the total numbers of NCD/HIV patients seen in the HIV clinic. In similar fashion, it is arguable that those with asymptomatic diseases may have been missed due to the lack of a policy of active NCDs screening. Both situations would underestimate comorbidity prevalence.

Disease misclassification is a possibility considering that the bulk of diagnoses and on-going care were nurse-driven. Our study did not seek to validate clinical diagnoses. We merely enumerated the diagnosed NCD cases. Non-infectious chronic lung diseases, for example, may have been misdiagnosed and treated as pulmonary TB. Lastly, we used routine data which is not usually collected with the rigour required for research purposes. Though the data were sufficiently complete allowing us to answer our research question, more detailed data would have enabled additional analyses, case ascertainment and more robust conclusions. Lastly, inclusion of an HIV uninfected age- and gender-matched comparison group would have afforded insights into the putative roles of HIV and ART as drivers of NCDs.

## Conclusion

In the light of these, as well as findings from other studies within and without SSA, it is strongly recommended that a policy of active screening for NCDs, especially hypertension and T2DM, be adopted as part of routine HIV care. Due to severe resource constraints in most SSA care settings, targeted screening, guided by age and gender for example, may be proposed. As the HIV epidemic increasingly overlaps with the growing problem of NCDs, demands for life-long care will multiply in SSA. It is high time to consider how the infrastructure that developed as a response to HIV—voluntary testing and point-of-care testing technologies, decentralization of treatment to primary care, public engagement and resource mobilisation, behavior change and social marketing strategies, among others—can be adapted and simultaneously deployed in the fight against NCDs.

Task shifting of HIV care in SSA from doctors to nurses and other less skilled cadres allowed the rapid scaling of treatment delivery in the face of severe human capacity constraints. The successes notwithstanding, the training needs of these front line cadres may not have kept pace with an evolving epidemic and its emerging problems. Beyond the burden of NCDs in HIV, huge gaps exist in our knowledge of diagnosis, treatment and outcomes of these comorbidities within the SSA context. This is in turn presents urgent priorities for research.

We have shown that, in keeping with reports from HICs, NCD comorbidities are emerging as a significant and growing challenge in HIV care in SSA. The first from Zimbabwe, a high HIV burden country, our report contributes to this growing body of knowledge of HIV and non-communicable diseases comorbidity. We also highlighted the knowledge gaps peculiar to our sub-Saharan African setting requiring research. Lastly, if the gains made in the fight against HIV are to be preserved, there is an urgent need to integrate NCD screening and care into HIV programmes.

## Abbreviations

NCD: non-communicable disease
HIV: human immunodeficiency virus
OLD: obstructive lung disease
WHO: World Health Organization
T2DM: type 2 diabetes mellitus
SSA: Sub-Saharan Africa
TB: tuberculosis
